# Phytohormones: the chemical language in *Magnaporthe oryzae*-rice pathosystem

**DOI:** 10.1080/21501203.2018.1483441

**Published:** 2018-06-12

**Authors:** Shulin Zhang, Yi Zhen Deng, Lian-Hui Zhang

**Affiliations:** Integrative Microbiology Research Centre, South China Agricultural University, Guangzhou, China; Guangdong Province Key Laboratory of Microbial Signals and Disease Control, South China Agricultural University, Guangzhou, China; State Key Laboratory for Conservation and Utilization of Subtropical Agro-Bioresources, South China Agricultural University, Guangzhou, China

**Keywords:** Fungal-plant interaction, phytohormones, defense response, inter-kingdom communication

## Abstract

Phytohormones (also named as plant hormones) are chemicals produced by plants in order to modulate various aspects of plant development, stress responses and defence. Recent studies revealed that fungi can also produce phytohormones or phytohormone-mimiking molecules, while it remains poorly understood about the details in the role and regulatory mechanism of such fungal produced phytohormonal molecules in plant-fungus interactions. The rice-blast fungus *Magnaporthe oryzae* imposes a great threat to global food security. Intensive investigation has been conducted to elucidate *M. oryzae* pathogenicity and rice (*Oryza sativa L*.) defense mechanism against blast disease, in order to provide theoretical basis and/or identify potential target(s) for developing novel disease control strategies, as well as for breeding of resistance varieties. Phytohormones have been demonstrated to play conserved and divergent roles in fine-tuning the balance of rice growth and immunity towards *M. oryzae*. Meanwhile, *M. oryzae* evolved elaborate strategy to manipulate the rice phytohormones metabolism, or even directly produce and secrete phytohormones, during their invasion process. In this review, we discuss the chemical communication in term of phytohormones in *M. oryzae*-rice pathosystem.

## Introduction

Plants dynamically response to environmental stimuli, including pathogen invasion. A sophisticated coevolution is indicated in parasitic association between fungi and plants, involving mutual perceptions and reactions (Jones and Dangl 2006). At the initial stage of infection, plants are able to recognise the molecular pattern unique to fungal pathogens (e.g. long-chain chitins in the fungal cell wall), generally named as pathogen-associated molecular pattern (PAMP), by its innate immune system and thus trigger a defense response called PAMP-Triggered Immunity (PTI) (Chisholm et al. 2006; Jones and Dangl 2006). To overcome plant PTI, pathogens secrete effector proteins into the apoplastic space of host cells or enter host cells, and suppress plant immune perception, eventually facilitate pathogen colonisation of host. Pathogen effectors could be recognised by host plant resistance proteins and trigger a second layer of plant innate immunity, effector-triggered immunity, to halt disease progress (Jones and Dangl 2006; Wang et al. 2014; Kachroo et al. 2017). In this competing relationship, phytohormones produced by plants serve as one of plant defence mechanisms against fungal invasion, while fungal pathogens have developed multiple strategies to disrupt plant phytohormones biosynthesis or signaling (Kazan and Lyons 2014; Chanclud and Morel 2016). More intriguingly, recent research revealed that fungi also produce some phytohormones, or metabolites mimicking phytohormones, likely to alter host physiology for their own benefit (Chanclud and Morel 2016).

Currently, eight types of phytohormone have been well established and their physiological roles in plant growth, development, abiotic and biotic stress resistance have been well-documented. These eight types of phytohormones include auxins (indole-3-acetic acid, IAA) (Austin et al. 2002; Azevedo et al. 2002), cytokinins (CKs) (Jiang et al. 2013), brassinosteroids (BRs) (Nolan et al. 2017), abscisic acid (ABA) (Hauser et al. 2017), gibberellins (GAs) (Singh 2002), salicylic acid (SA) (Boatwright and Pajerowska-Mukhtar 2013), jasmonates (JAs) (Wasternack and Hause 2013) and ethylene (ET). In *Arabidopsis thaliana*, SA, JAs and ET are mainly involved in plant defense response (Lopez et al. 2008; Robert-Seilaniantz et al. 2011; Kazan and Lyons 2014; Patkar and Naqvi 2017). Particularly, SA mediates plant resistance towards biotrophic or semibiotrophic pathogens, and is involved in the systemic acquired resistance (SAR) in which a pathogenic attack on one part of the plant induces resistance in other parts; JAs and ET mediate resistance to necrotropic plant pathogens (Broekaert et al. 2006; Pieterse et al. 2012). Auxins, CKs, BRs, ABA and GA are mainly involved in regulating various aspects of plant growth and development, including root and shoot growth, flowering, leaf senescence, fruit ripening, seeds dormancy and germination, etc, but they may also participate in disease resistance through SA, JAs or ET signaling regulation (Jiang et al. 2013; Verma et al. 2016; Nolan et al. 2017).

*Magnaporthe oryzae* is the number one of the top 10 fungal pathogens, based on its scientific/economic importance, in a survey with fungal pathologists (Dean et al. 2012). The ascomycete fungus *M. oryzae* causes rice-blast disease, the most destructive disease of rice that leads to 10–30% yield losses each year worldwide and thus poses great thread to global food security (Talbot 1995; Dean et al. 2012; Kazan and Lyons 2014). Therefore, intensive investigation has been conducted on *M. oryzae*-rice interaction for elucidation of fungal pathogenicity as well as rice defense strategies. In this review, we discuss the recent advances on fungal manipulation of, and fungal-derived, auxin, CKs, ABA, and JAs, in *M. oryzae*-rice pathosystem.

## Auxin in *M. oryzae*-rice pathosystem

Limited reports are available to address the role of auxin in *M. oryzae*-rice pathosystem. It was observed that *M. oryzae* infection repressed auxin signal pathway in uninfected leaves of rice, by down-regulating auxin responsive genes *ARF1* and *IAA9*, which is likely responsible for reduced growth of uninfected leaves and as SAR to restrict *M. oryzae* infection (Jiang et al. 2017). Overexpression of auxin conjugation enzyme OsGH3.1 caused less susceptible to *M. oryzae* due to down-regulation of auxin response and induction of resistance gene (Hagen and Guilfoyle 2002; Mukesh Jain and Tyagi 2006; Domingo et al. 2009). In contrast, accumulation of auxin resulted from infection of rice roots by root knot nematode leads to enhanced susceptibility of rice leaves to *M. oryzae* (Kyndt et al. 2017). Therefore, rice may suppress its own auxin response upon perception of *M. oryzae* infection, in order to halt its growth and induce defense response. To counteract such hormonal change in host, *M. oryzae* could produce IAA in its hyphae and conidia (Jiang et al. 2013), probably to trick the rice to grow but not to defense. It is worth further studying on such fungal IAA production, and likely, secretion, to elucidate its biosynthesis (if any) and function in *M. oryzae* physiology.

Overall, recent studies demonstrated that auxin signaling pathway positively regulates rice growth while negatively regulates blast resistance; therefore, manipulation of it via down-regulating genes involved in inhibition of auxin response (promoting auxin response and plant growth) is favourable for *M. oryzae* infection.

## CKs in *M. oryzae*-rice pathosystem

Besides auxin, various CKs were also produced by *M. oryzae* in its hyphae and condia (Chanclud et al. 2016). *M. oryzae CKS1* gene, encoding a putative tRNA-IPT protein, was shown to be essential for CK biosynthesis and specifically required for in planta growth and virulence (Chanclud et al. 2016). CKs were also shown to be released into the culture medium after production (Chanclud et al. 2016). CKs may facilitate nutrient translocation for the blast fungus (Walters et al. 2008; Chanclud et al. 2016). On the other hand, *M. oryzae* infection triggered CK accumulation in rice seedlings which activated CK signaling and subsequently induced expression of resistance gene *OsPR1b* and *PBZ* to activate rice resistance against blast. Such CK-mediate blast resistance was synergistically regulated by SA signaling pathway (Jiang et al. 2013). Consistently, co-treatment of rice leaf blades with CKs and SA strongly induced the expression of the defense genes *PR1b* and *PBZ1*, whereas treatment with either one alone only slightly increased their expression levels (Jiang et al. 2013).

In summary, both the rice and the blast fungus are able to produce CKs. *M. oryzae* produces and secretes CKs to facilitate its own nutrient translocation, while such signal molecules was proposed yet not verified, to be perceived by the host rice and triggers plant CKs signaling pathway to regulate defense response, together with SA signaling pathway. It remains unclear as how the fungal CKs sensed by the host and which signal transduction pathway is responsible for inducing plant CKs response. Similar as auxin function, mutual manipulation of this plant growth hormone may represent a mechanism for balancing plant growth, especially cell division and/or cell death, and defense reaction in rice.

## ABA in *M. oryzae*-rice pathosystem

It has been shown that overproduction of ABA in plants may have an adverse effect on disease resistance, due to suppression of SAR mediated by SA, JA and ET signaling pathway (Anderson 2004; Ton et al. 2009; Nahar et al. 2012). Reduction of ABA production or disruption of ABA signaling in rice enhanced resistance to rice-blast disease (Yazawa et al. 2012). Conversely, ABA treatment of rice seedlings resulted in rice susceptibility towards incompatible and compatible *M. oryzae* race (Jiang et al. 2017). Therefore, fungal-derived ABA could potentially act as a virulence factor.

Some fungal pathogens were found to produce ABA, mainly via mevalonate pathway (Oritani and Kiyota 2003; Siewers et al. 2006), which is different from the ABA biosynthesis pathway in plants. A gene cluster including *BcABA1, BcABA2, BcABA3* and *BcABA4* involved in ABA synthesis was identified in ascomycete *B. cinerea* (Siewers et al. 2006). In *M. oryzae*, ABA was detected during vegetative growth and spores formation stages (Spence and Bais 2015). Knowledge about ABA biosynthesis is limited in *M. oryzae*. Three *ABA* gene homologs (*MoABA1, MoABA2* and *MoABA4*) and an ABA G-protein couple receptor were identified in *M. oryzae* (Spence et al. 2015). Deletion of *MoABA4* gene resulted in loss of pathogenicity, indicating that ABA production may be crucial for *M. oryzae* pathogenicity (Spence et al. 2015). *M. oryzae* was able to up-regulate the rice *NCED3* gene (for rice ABA biosynthesis) expression, suggesting that it may stimulate ABA synthesis in rice to facilitate its own pathogenicity and subvert host resistance (Spence et al. 2015).

Overall we draw a conclusion that in the *M. oryzae*-rice interaction, ABA plays a dual role in disease severity by suppressing plant resistance and accelerating pathogenesis in the fungus itself.

## JAs in *M. oryzae*-rice pathosystem

Rice produces low-molecular-weight antimicrobial compounds known as phytoalexins, mainly composed of diterpenoids and a flavonoid. Jasmonate isoleucine (JA-Ile) is a bioactive form of JA, and its level increases in response to blast infection. Endogenous JA-Ile is involved in blast-resistance mainly through facilitating production of the flavonoid phytoalexin, sakuranetin (Miyamoto et al. 2016). JA-Ile synthesis was catalysed by JA-Ile synthase. Recent study showed that two JA-Ile synthases OsGH3.5 (OsJAR1) and OsGH3.3 (OsJAR2) are functional in JA-Ile production in rice. Particularly, expression of *OsJAR1* was associated with the accumulation of JA-Ile after blast infection (Wakuta et al. 2011), indicating a role in blast-resistance via JA-signaling.

On the other hand, *M. oryzae* managed to defeat rice defense by manipulating rice JA-signaling pathway. One example is that *M. oryzae* specifically induced the expression of rice miR319, whose target gene encodes a transcription factor OsTCP21. OsTCP21 is a positive regulator of the rice defense response against the blast disease, likely via inducing JA synthesis genes *LOX2* and *LOX5* (Zhang et al. 2018). Therefore, *M. oryzae* is able to reduce rice JA level by suppressing JA synthesis via inducing rice miR319. Also, *M. oryzae* is able to modify rice JA molecule to an inactive form 12-OH JA, by a monooxygenase, and thus subvert host immunity (Patkar and Naqvi 2017).

In summary, rice induces JA-Ile synthesis in response to blast infection, while *M. oryzae* suppresses JA synthesis via a rice miRNA pathway, and likely convert rice JA to 12-OH JA to inactive JA-mediated SAR.

## Concluding remark

**Figure 1.**
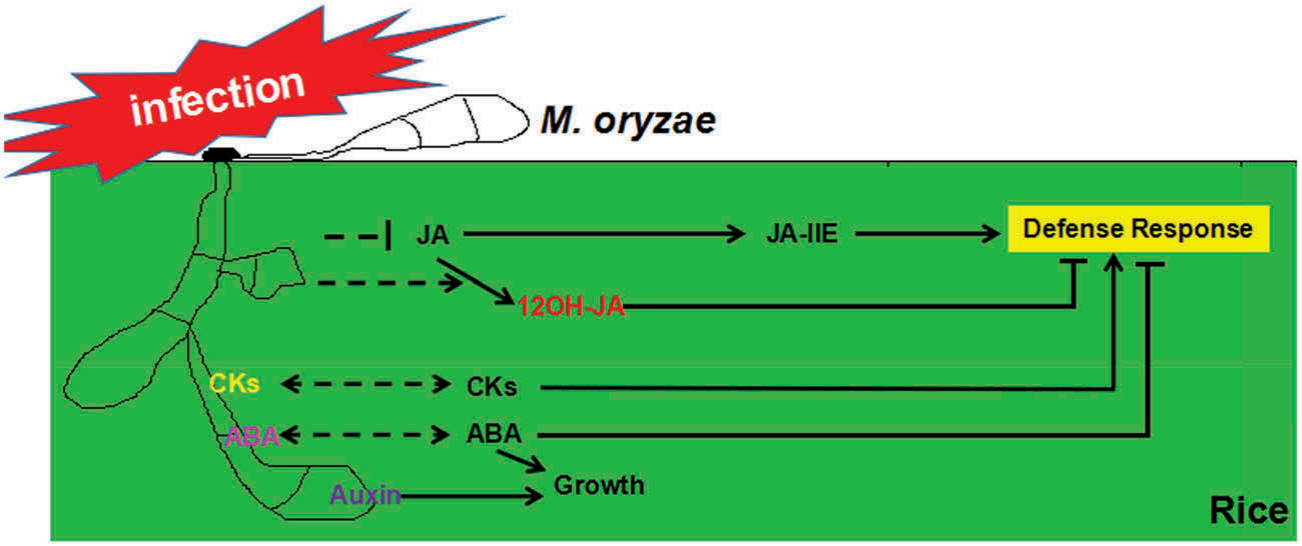
**Schematic summary of inter-kingdom communication between *M. oryzae* and rice using phytohormones**. *M. oryzae* produces (and like secretes) auxin, CKs and ABA during its infection on rice. Both auxin and ABA could promote rice growth while suppress its defense agains blast-disease. *M. oryzae* may use CKs for nutrient translocation, but meanwhile CKs signaling in rice may trigger defense response. M. oryzae is able to suppress rice JA biosynthesis and simultaneously convert JA to 12-OH JA, to interfere with rice defense response mediated by JA-signaling pathway.

Phytohormones are small molecules produced by plants to regulate growth and development in response to various physiological or environmental stimuli, thus simulate a language used by plants for better communication among different parts. Pathogenic fungi, that deploy nutrients from host for their own survival, learn to speak this phytohormone language during their co-evolution with host plants. In this review, we use *M. oryzae*-rice pathosystem to demonstrate how phytohormones are involved in such inter-kingdom communication (Figure 1). *M. oryzae* is able to synthesise (and likely secrete) phytohormonal molecules including auxin (IAA), ABA and various CKs, and also able to induce rice ABA synthesis. *M. oryzae* could reduce rice JA synthesis, and convert JA to 12-OH JA synthesis instead of production of an active blast-resistance JA derivative, JA-Ile. In this review we individually discussed each type of phytohormone in *M. oryzae*-rice interaction, but in actual scheme the phytohomone signaling pathways are in a complex network with multiple crosstalk with each other. So far it has not been reported on whether other types of phytohormones (SA/ET/BR etc) could be derived from *M. oryzae*, although such possibility could not be ruled out. By this review we would like to emphasise that phytohormones are not just plant growth regulators, but also a chemical language used between plants and fungi, for efficient inter-kingdom (competitive) communication.
